# The BR signaling pathway regulates primary root development and drought stress response by suppressing the expression of *PLT1* and *PLT2* in *Arabidopsis thaliana*

**DOI:** 10.3389/fpls.2023.1187605

**Published:** 2023-06-27

**Authors:** Zhiying Zhao, Shuting Wu, Han Gao, Wenqiang Tang, Xuedan Wu, Baowen Zhang

**Affiliations:** ^1^ Ministry of Education Key Laboratory of Molecular and Cellular Biology; Hebei Research Center of the Basic Discipline of Cell Biology, Hebei Collaboration Innovation Center for Cell Signaling and Environmental Adaptation, Hebei Key Laboratory of Molecular and Cellular Biology, College of Life Sciences, Hebei Normal University, Shijiazhuang, China; ^2^ Institute of Crop Sciences, Chinese Academy of Agricultural Sciences, Beijing, China

**Keywords:** PLT1, PLT2, brassinosteroids, drought, root

## Abstract

**Introduction:**

With the warming global climate, drought stress has become an important abiotic stress factor limiting plant growth and crop yield. As the most rapidly drought-sensing organs of plants, roots undergo a series of changes to enhance their ability to absorb water, but the molecular mechanism is unclear.

**Results and methods:**

In this study, we found that PLT1 and PLT2, two important transcription factors of root development in *Arabidopsis thaliana*, are involved in the plant response to drought and are inhibited by BR signaling. PLT1- and PLT2-overexpressing plants showed greater drought tolerance than wild-type plants. Furthermore, we found that BZR1 could bind to the promoter of *PLT1* and inhibit its transcriptional activity *in vitro* and *in vivo*. *PLT1* and *PLT2* were regulated by BR signaling in root development and *PLT2* could partially rescue the drought sensitivity of *bes1-D*. In addition, RNA-seq data analysis showed that BR-regulated root genes and PLT1/2 target genes were also regulated by drought; for example, *CIPK3*, *RCI2A*, *PCaP1*, *PIP1;5*, *ERF61* were downregulated by drought and PLT1/2 but upregulated by BR treatment; *AAP4*, *WRKY60*, and *AT5G19970* were downregulated by PLT1/2 but upregulated by drought and BR treatment; and *RGL2* was upregulated by drought and PLT1/2 but downregulated by BR treatment.

**Discussion:**

Our findings not only reveal the mechanism by which BR signaling coordinates root growth and drought tolerance by suppressing the expression of PLT1 and PLT2 but also elucidates the relationship between drought and root development. The current study thus provides an important theoretical basis for the improvement of crop yield under drought conditions.

## 1 Introduction

With global warming, reduced rainfall, and other factors, drought has become one of the most serious abiotic stresses. Drought causes a series of impairments to the physiological, biochemical, and metabolic processes of plants, thereby causing plant growth retardation, cell damage, declines in crop yield and quality, and even plant death (Blum, 2009; Hu and Xiong, 2014; Lorts and Lasky, 2020). Therefore, studying the mechanism by which plants respond to drought is essential for the enhancement of plant drought tolerance and the improvement of crop yield.

Being sessile, plants have root systems that enable them to anchor themselves in the soil and obtain nutrients and water from their environments. Thus, the roots are important organs in the initial response to drought stress (Koevoets et al., 2016; Dinneny, 2019; Kim et al., 2020; Qin et al., 2022). When the roots of plants sense drought stress, their root tip stem cells, meristems, and vascular tissues coordinate with each other to affect the morphological structure of the roots and ultimately increase the area or length of the roots in order to enhance the root water absorption capacity (Gupta et al., 2020). Recent studies have found that drought, if not too severe, enhances root growth, which allows plants to absorb water from deeper soils, and roots with smaller diameters and greater lengths have larger surface areas in contact with water (Xu et al., 2013; Gupta et al., 2020; Kim et al., 2020). However, whether the components regulating root length are involved in the drought stress response is unclear. Furthermore, the molecular mechanism by which roots perceive drought and its downstream signaling pathway still needs further exploration.

Root length is determined by cell number and cell size. The root apical meristem produces a series of cells that can elongate and differentiate after multiple divisions. Therefore, the activity of the meristem is a key factor determining root length (Beemster and Baskin, 1998; Petricka et al., 2012). The PLETHORAs (PLTs), a class of transcription factors containing the APETALA2/ETHYLENE RESPONSE FACTOR (AP2/ERF) domain, play an important role in root development (Aida et al., 2004; Galinha et al., 2007; Mähönen et al., 2014). Among them, PLT1 and PLT2 are highly homologous. When the two genes encoding these proteins are mutated, the root length of the plant is severely shortened, and the number of apical meristem cells is reduced; thus, the activity of PLT1 and PLT2 is crucial for the maintenance of the apical meristem (Aida et al., 2004; Galinha et al., 2007). However, whether and how these two genes participate in the drought stress response remains unknown.

Brassinosteroids (BRs) play important roles in several plant life processes, including cell elongation and division, photomorphogenesis, plant senescence, and responses to various biological and abiotic stresses (Clouse, 1998; Müssig et al., 2003; Clouse, 2011; Yang et al., 2011). BR can be recognized by the membrane receptor BRASSINOSTEROID INSENSITIVE 1 (BRI1), which promotes the reciprocal phosphorylation of BRI1 with the coreceptor BRI1-ASSOCIA TED RECEPTOR KINASE 1 (BAK1/SERK3), inhibits the activity of the negative regulator GSK3/Shaggy-like kinase (BRINSENSITIVE2, BIN2) and promotes the degradation of BIN2 (Vert and Chory, 2006; Peng et al., 2008). Thus, BIN2 is unable to phosphorylate the downstream transcription factors BRASSINAZOLE RESISTANT 1 (BZR1) and BRI1-EMS-SUPPRESSOR 1 (BES1). Phosphorylated BZR1/BES1 is dephosphorylated by PROTEIN PHOSPHATASE 2A (PP2A) and binds to downstream target genes to regulate various growth and developmental processes in plants (Sun et al., 2010; Tang et al., 2011; Yu et al., 2011; Kono and Yin, 2020).

BRs are important participants in both root development and drought stress response (González-García et al., 2011; Chaiwanon and Wang, 2015; Nolan et al., 2017; Ye et al., 2017). BR can influence the size of the root meristem by regulating the self-renewal ability of the quiescent center (QC) and the expression of cell cycle-related genes (Vilarrasa-Blasi et al., 2014; Planas-Riverola et al., 2019). Interestingly, depending on the plant species and the BR signaling- or biosynthesis-related genes studied, BR has been found to play both positive and negative roles in regulating plant drought response (Sahni et al., 2016; Ye et al., 2017; Fàbregas et al., 2018; Cui et al., 2019; Nie et al., 2019). The paradoxical results observed in these studies suggest that BR signaling regulates plant drought response *via* multiple mechanisms. Therefore, identifying the tissue- and plant-specific drought-regulating components, such as root-specific drought-regulating proteins, that can be controlled *via* the BR signaling pathway is essential to understanding the role of BR signaling in regulating plant drought resistance.

In this study, to reveal the relationship between drought stress and plant root growth and development, we first explored whether PLT1 and PLT2 are involved in the drought response. We found that the drought tolerance of *PLT1-* and *PLT2*-overexpressing plants was significantly enhanced compared with that of the wild type, indicating that these genes might involved in the response to drought. We also found that BZR1 could directly bind to the promoter of *PLT1* and repress its expression. BR application reduced the expression levels of *PLT1* and *PLT2* in roots, but the *PLT2*-overexpressing plants were less BR-sensitive than the wild-type plants during primary root growth. In contrast, the double mutant *plt1plt2* had enhanced sensitivity to BR, suggesting that PLT1 and PLT2 are regulated by the BR signaling pathway and function redundantly. Genetic experiments revealed that *PLT2* overexpression suppressed the drought-sensitive phenotype of *bes1-D*, suggesting that PLT2 acts downstream of the BR signaling pathway to regulate the drought response. Further RNA-seq analysis showed that some drought- and BR-related genes were coregulated by PLT1/2. Our study not only reveals that the root growth regulatory factors PLT1 and PLT2 can regulate the drought stress response but also further clarifies that BR participates in root development and the drought stress response by inhibiting the expression of *PLT1* and *PLT2*.

## 2 Materials and methods

### 2.1 Plant materials and growth conditions

The Arabidopsis mutant and transgenic plants used in this study, including *bzr1-1D*, *bes1-D*, *plt1 plt2*, *pPLT1:PLT1-YFP/Col-0*, and *pPLT2:PLT2-YFP/Col-0*, are in the Col-0 background and have been reported previously (An et al., 2018). To check *bes1-D* and *PLT2-OX/bes1-D* in different backgrounds, we performed MspI Digest and western blot. The plants were grown at 22°C and 55% relative humidity under long-day conditions (16 h of light at 100 μmol/m^2^s, followed by 8 h of darkness) either in a growth chamber or in a growth room equipped with T5 4000K fluorescent tubes (Philips). Double or triple mutants generated by genetic crossing and F3-segregated homozygous plants were used for phenotypical or biochemical analysis.

### 2.2 BR treatment and drought treatment

The different concentrations of eBL treatment were described previously (Zhao et al., 2021). 10 days Col-0 seedlings were immersed in 1 μM eBL for 0-9 h, and roots were placed into liquid nitrogen immediately after treatment. 14 days Col-0, *bzr1-1D*, and *bes1-D* seedlings were grown with 0.25 μM PCZ, and roots were placed into liquid nitrogen immediately after treatment. Then, RNA was extracted to detect *DWF4* (BR synthesis gene), *PLT1*, and *PLT2* expression. For the drought-stress treatments, the seedlings with uniform growth were either watered as usual (control) or stopped watering for 18 days and rewatered 12 days before taking photos for phenotype comparison. Each pot is considered as one biological replicate. In general, at least 10 biological replicates collected from three to four independent experiments were used for survival rate quantification.

### 2.3 qRT-PCR analysis

First-strand cDNA was synthesized from approximately 1 µg total RNA using M-MLV Reverse Transcriptase (Takara Bio Inc., Otsu, Japan). Quantitative real-time PCR was performed according to the standard protocol using a Bio-Rad CFX Connect real-time PCR machine (Bio-Rad Laboratories, Hercules, CA, United States) using the SYBR Premix Ex TaqTM system (Takara Bio Inc.). The primers used are listed in **Supplementary Table S5**. *UBC30* as an internal reference. Averaged value from at least three biological replicates was presented.

### 2.4 Electrophoretic mobility shift assays

EMSA was performed using recombinant MBP-BZR1 proteins purified from E. coli and biotin-labeled double-stranded oligonucleotide probes containing BRRE sequences chosen from the *PLT1* promoter. Probe Primers used are listed in **Supplementary Table S5**. EMSAs was performed as described by Zhao et al. (2021) (Zhao et al., 2021).

### 2.5 Transient gene expression assay

The wild-type and mutation forms of the 1884 bp promoter sequences of *PLT1* were incorporated into *pGreenII 0800-LUC* dual luciferase vector (Hellens et al., 2005) using LR Clonase (Invitrogen). *Arabidopsis thaliana* protoplast transformations were performed according to the methods described by Yang et al. (2016) (Yang et al., 2016). Relative promoter activity was calculated by dividing the *PLT1* promoter-driven firefly luciferase signal by the *35S* promoter-driven renilla signal which serves as the internal normalization control. The experiment has been repeated at least three times with similar results.

### 2.6 Statistical analysis

A one-way analysis of variance (ANOVA) or two-way ANOVA with Tukey’s honestly significant difference test was performed to evaluate the differences across sample groups or treatments. Statistically significant differences are indicated by different lowercase letters (*P* < 0.05, ANOVA test), one asterisk (*P* < 0.05, Student’s *t*-test), or two asterisks (*P* < 0.01, Student’s *t*-test).

## 3 Results

### 3.1 PLT1 and PLT2 play positive roles in the drought response in Arabidopsis

**Figure 1:**
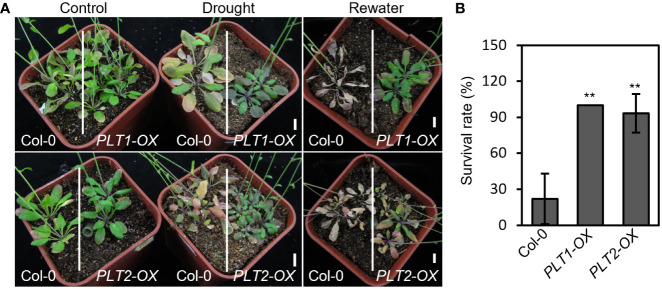
Overexpression of *PLT1* and *PLT2* enhances drought tolerance in Arabidopsis. **(A)** The phenotypes of wild type (Col-0), *PLT1-OX*, and *PLT2-OX* plants that were subjected to well-watered (control), drought, and rewater conditions. **(B)** Quantitation of the survival rates of the drought-treated plants shown in **(A)** after 12 days of re-watering, n≥40. Error bars indicate mean ± SD. Statistically significant differences are indicated by asterisks (Student’s *t*-test, *p < 0.05, **p < 0.01).

To investigate whether root-specific regulatory components play an important role in the response to drought stress, we performed drought stress experiments on mutants and on plants overexpressing genes that regulate root growth and development. We found that the plants overexpressing *PLT1* and *PLT2* (*PLT1-OX* and *PLT2-OX*), which are important transcription factors regulating root growth and development, showed significantly higher survival rates and better drought resistance after drought treatment than the wild-type Col-0 plants (**Figure 1**), suggesting that PLT1 and PLT2 positively regulate the plant response to drought.

### 3.2 BZR1 binds to the promoter of *PLT1* and inhibits its expression

**Figure 2:**
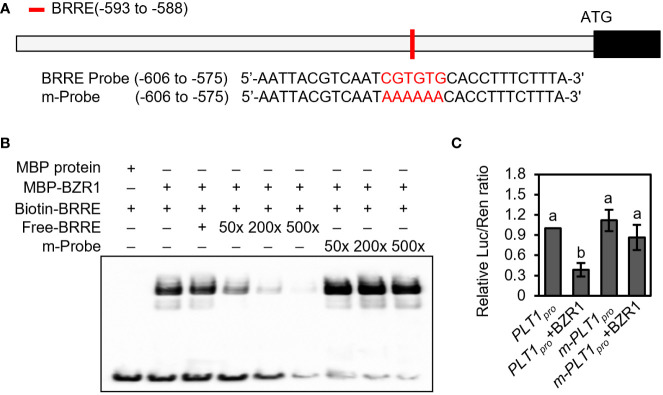
BZR1 binds to the promoter of *PLT1* and represses its expression. **(A)** Schematic diagram of the *PLT1* promoter and probe sequences. The red box represents the BRRE motif, and the red letters represent the BRRE sequence and the mutant sequence. **(B)** BZR1 bound the BRRE *cis*-element in the *PLT1* promoter. EMSA was performed using recombinant proteins *in vitro* as described in the Materials and Methods. **(C)** BZR1 represses *PLT1* expression *in vivo*. Arabidopsis leaf protoplasts were co-transformed with *PLT1_pro_:luciferase* (*PLT1_pro_*) or *PLT1_pro_mBRRE:luciferase* (*m*-*PLT1_pro_*) vector plasmids and *35S_pro_ : BZR1-GFP* (BZR1) or *35S_pro_ : GFP* vector plasmids, and the relative luciferase activity was measured by Luc/Ren ratio, n=3. Error bars indicate mean ± SD. Statistically significant differences are indicated by different lowercase letters (one-way ANOVA, *p* < 0.05).

The BR signaling pathway can regulate both root development and plant drought response, but whether BR regulates the drought stress response by regulating root-specific components remains unclear. Our previous findings revealed that the BZR1 homolog in foxtail millet, *SiBZR1*, recognizes and binds to the BR response element (BRRE) in the promoter region of the *PLT1* homolog *SiPLT-L1* and downregulates its expression to affect root development (Zhao et al., 2021). However, transcriptional activity assays *in vivo* and genetic experiments had yet to be completed. To further characterize the mechanism, we analyzed the promoter sequence of *PLT1* in Arabidopsis and found that the 2000 bp promoter and 5’-UTR of *PLT1* contained a BRRE (CGTGTG) *cis-*element (**Figure 2A**).

Next, we performed an EMSA, which showed that MBP-BZR1 was able to bind to DNA probes containing the BRRE of the *PLT1* promoter *in vitro*. When unlabeled probes were added to the reaction system, the binding of biotin-labeled probes to MBP-BZR1 was reduced, and the binding of biotin-labeled probes to MBP-BZR1 was weakened with increased concentrations of unlabeled probes (**Figure 2B**). When the BRRE sequence was mutated to AAAAAA, the mutant probe exhibited a significantly reduced ability to compete with the biotin-labeled probe (**Figure 2B**). This suggests that BZR1 can specifically recognize and bind to the BRRE *cis*-element of *PLT1*. In transient transformation experiments in Arabidopsis protoplasts, we further showed that BZR1 was able to repress the expression of *pPLT1:Luciferase*, and mutating the BRRE of the *PLT1* promoter significantly attenuated the repressive effect (**Figure 2C**). These results suggest that BZR1 binds to the BRRE-acting element on the *PLT1* promoter and represses *PLT1* gene expression in Arabidopsis and that the regulatory mechanism is conserved in the dicotyledonous plant Arabidopsis and the monocotyledonous foxtail millet. These results also indicate that BR may regulate Arabidopsis root growth and development through *PLT*-family genes.

### 3.3 PLT1 and PLT2 mediate BR-regulated primary root elongation

**Figure 3:**
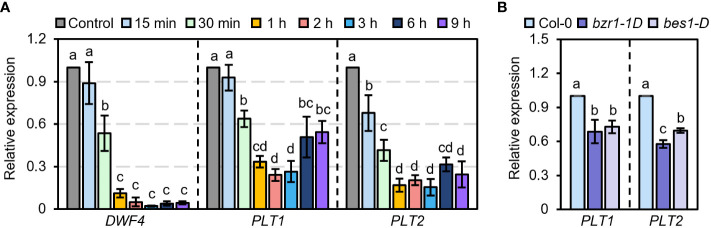
PLT1 and PLT2 are involved in the BR signaling pathway. **(A)** The relative expression levels of *DWF4, PLT1*, and *PLT2* in the roots of light-grown 10 days Col-0 plants treated with 1 µM eBL for the indicated time. **(B)** The relative expression levels of *PLT1* and *PLT2* in the roots of Col-0, *bzr1-1D*, and *bes1-D* plants that were grown in 0.25 μM PCZ for 14 days, n=3. Error bars indicate mean ± SD. Statistically significant differences are indicated by different lowercase letters (two-way ANOVA, *p* < 0.05).

PLT1 and PLT2 are two highly homologous genes that participate in the growth and development of plant roots in a functionally redundant manner (Aida et al., 2004). To determine whether BR are involved in PLT1- and PLT2-related regulation of root growth and development in Arabidopsis, we first explored whether PLT1 and PLT2 are involved in the response to BR. We treated wild-type Col-0 plants with BR at different times and examined the expression levels of *PLT1* and *PLT2* in the roots. We found that BR treatment for 30 min significantly suppressed the transcript levels of *PLT1* and *PLT2* and that the expression levels of *PLT1* and *PLT2* reached the lowest value after BR treatment for 1-3 h. When the treatment time was extended to 6-9 h, the transcript levels of *PLT1* and *PLT2* partially recovered but were still lower than those in the control group without BR treatment (**Figure 3A**). Similarly, the transcript levels of *PLT1* and *PLT2* were significantly lower in the BR gain-of-function mutants *bzr1-1D* and *bes1-D* than in Col-0 (**Figure 3B**). These results suggest that PLT1 and PLT2 are indeed regulated by the BR signaling pathway.

**Figure 4:**
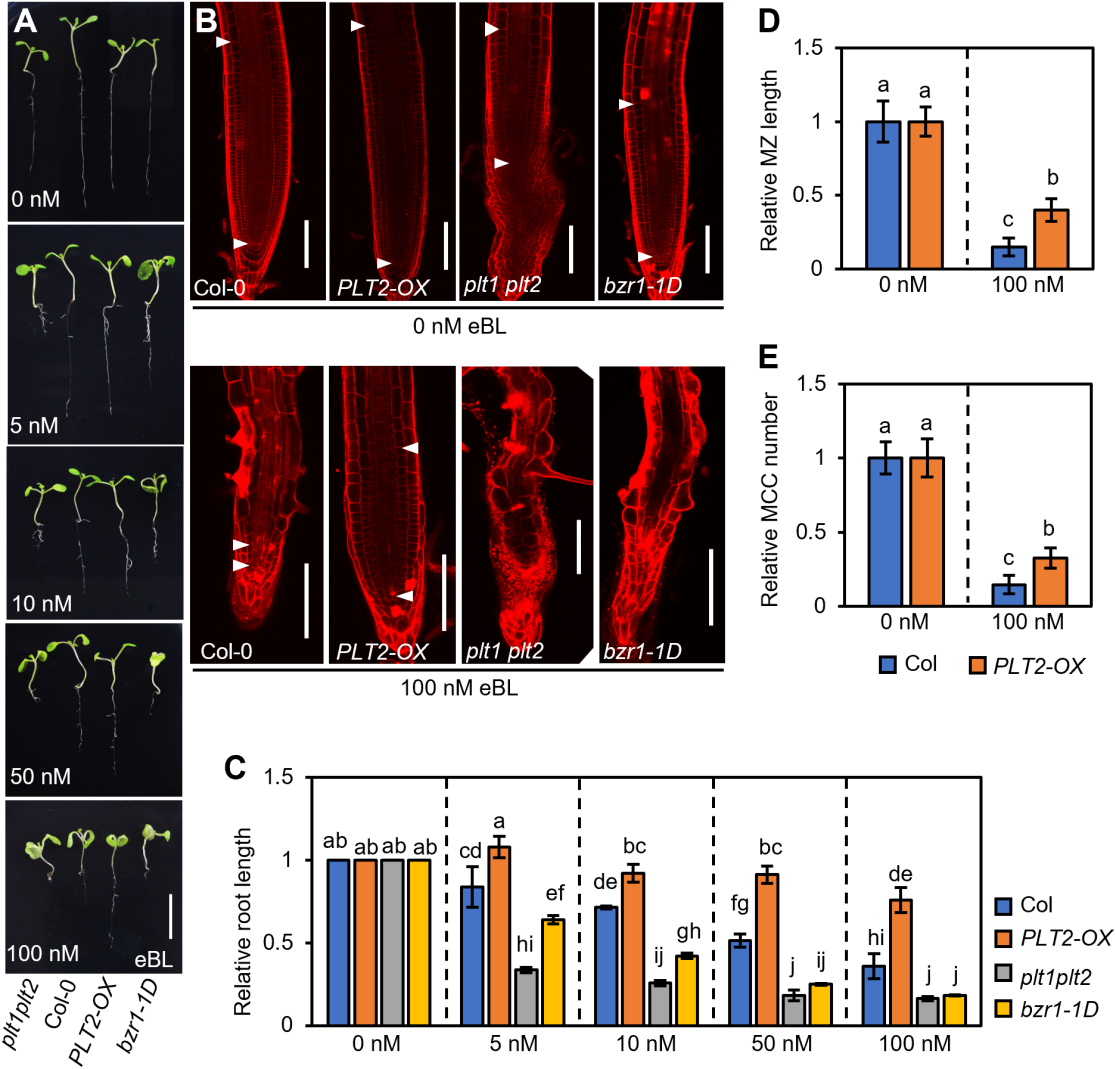
PLT1 mediates BR-regulated primary root elongation in Arabidopsis. **(A)** Root phenotypes of Col-0, *PLT2-OX*, *plt1plt2*, and *bzr1-1D* plants that were grown on vertical 1/2MS plates supplied with the indicated concentration of eBL for 7 days. Scale bar, 1 cm. **(B)** Propidium iodide (PI)-stained root meristems of the plants shown in **(A)** The white arrowheads mark the meristematic zone (MZ). Scale bar, 100 μm. **(C)** Quantitation of relative root length for the plants shown in **(A)**. **(D, E)** Quantitation of the relative MZ length **(D)** and relative meristematic cortex cell (MCC) number **(E)** for the plants shown in **(B)**, n>10. Error bars indicate mean ± SD. Statistically significant differences are indicated by different lowercase letters (two-way ANOVA, *p* < 0.05).

Since the expression of *PLT2-OX* was significantly higher than that of *PLT1-OX*, making it easier to observe (**Supplementary Figure S1**), we next selected *PLT2-OX* plants for subsequent experiments. To further determine the role of PLT1 and PLT2 in BR-mediated root growth and development, Col-0, *PLT2-OX*, *plt1plt2*, and *bzr1-1D* plants were grown in 1/2 MS medium containing different concentrations of epibrassinolide (eBL), and the root phenotypes of 7-day-old seedlings were observed (**Figure 4A**). Root elongation was promoted at low concentrations of eBL, but the root length of Col-0 plants gradually decreased as the eBL concentration increased, which was mainly due to shortening of the meristem and a decrease in the number of cortex cells in the meristem (**Figures 4B, C** **)**. This was consistent with results reported previously (González-Garcia et al., 2011; Zhao et al., 2021). Compared with Col-0 roots, *PLT2-OX* roots were significantly less sensitive to eBL. Under the same concentration of eBL, the root length of *PLT2-OX* was significantly greater than that of the wild type. Under 100 nM eBL growth conditions, the relative meristem length and the number of cortex cells in the meristem were also significantly higher than those of the wild type (**Figures 4B–E**). In contrast, the roots of *plt1plt2* and *bzr1-1D* had enhanced sensitivity to eBL, and they were significantly shorter than those of the wild type under the same conditions. Moreover, the *plt1plt2* and *bzr1-1D* root apical meristem cells were disordered under 100 nM eBL growth conditions, making them difficult to observe (**Figures 4B–E**). Taken together, these results suggest that PLT1 and PLT2 participate in the BR signaling pathway and that BZR1/BES1 inhibits root growth by suppressing *PLT1* and *PLT2* expression in Arabidopsis.

### 3.4 PLT1 and PLT2 participate in BR signaling-mediated drought stress

**Figure 5:**
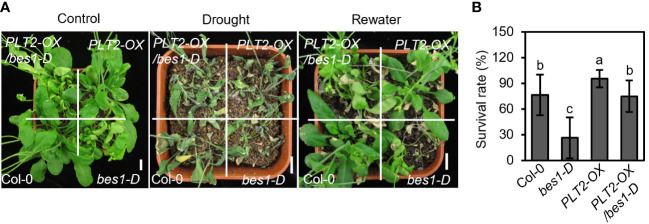
*PLT2-OX* enhances drought tolerance of *bes1-D*. **(A)** The phenotype of wild type (Col-0), *PLT1-OX*, *bes1-D*, and *PLT2-OX/bes1-D* plants that were subjected to well-watered (control), drought, and rewater conditions. **(B)** Quantitation of the survival rates of the drought-treated plants shown in **(A)** after 12 days of re-watering. n=11. Error bars indicate mean ± SD. Statistically significant differences are indicated by different lowercase letters (One-way ANOVA, *p* < 0.05).

BR also plays an important role in the response to drought, and BZR1/BES1 can inhibit root growth by repressing the expression of *PLT1* and *PLT2*, so we investigated whether BR regulates the drought response through a similar pathway. To answer this question, *PLT2-OX* plants were crossed with the BR gain-of-function mutant *bes1-D*, which is hypersensitive to drought (Ye et al., 2017), to obtain *PLT2-OX/bes1-D* plants (**Supplementary Figure S2**). The drought tolerance of related plants was then tested. Consistent with previous reports, the survival rate of *bes1-D* was significantly lower than that of Col-0 under drought conditions, while the survival rate of *PLT2-OX* was significantly higher than that of Col-0 (**Figure 5**). However, the survival rate of *PLT2-OX/bes1-D* was close to that of Col-0 under drought stress (**Figure 5**), this suggests that *PLT2* overexpression suppresses the drought-sensitive phenotype of *bes1-D* and that BES1 suppresses *PLTs* transcription expression partially contribute to regulate drought tolerance in Arabidopsis.

**Figure 6:**
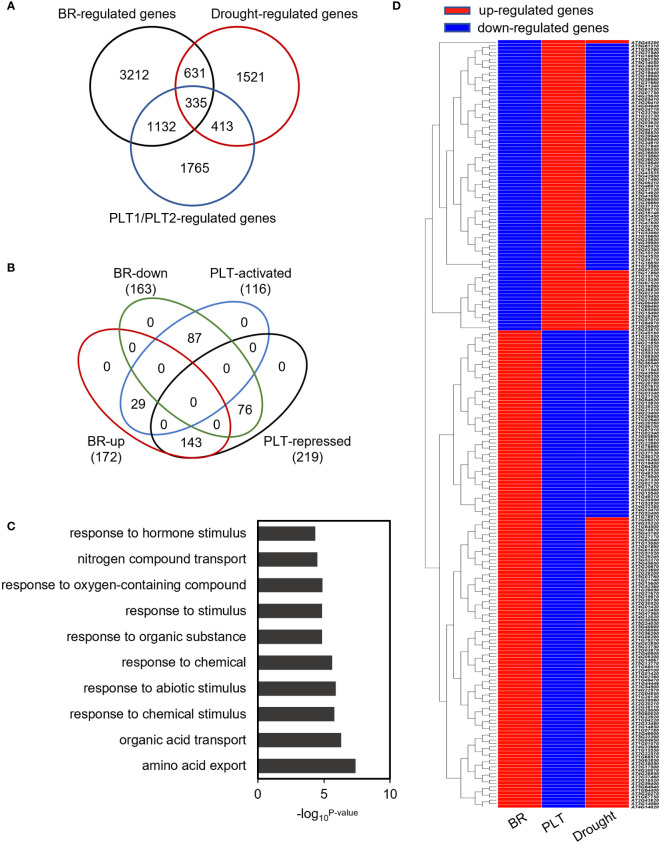
Co-regulated genes analysis of BR, PLT1, PLT2, and drought. **(A)** Venn diagrams of BR-regulated genes, drought-regulated genes, and PLT1/2 target genes. **(B)** Venn diagrams of BR up or down and PLT1-PLT2 activated or repressed of 335 regulated genes. **(C)** GO classification of the 230 regulated genes. **(D)** The expression heatmap showing the 230 regulated genes antagonistically regulated in BR, PLT1-PLT2, and drought. Red indicates up or activated genes, and blue indicates down or repressed genes.

**Figure 7:**
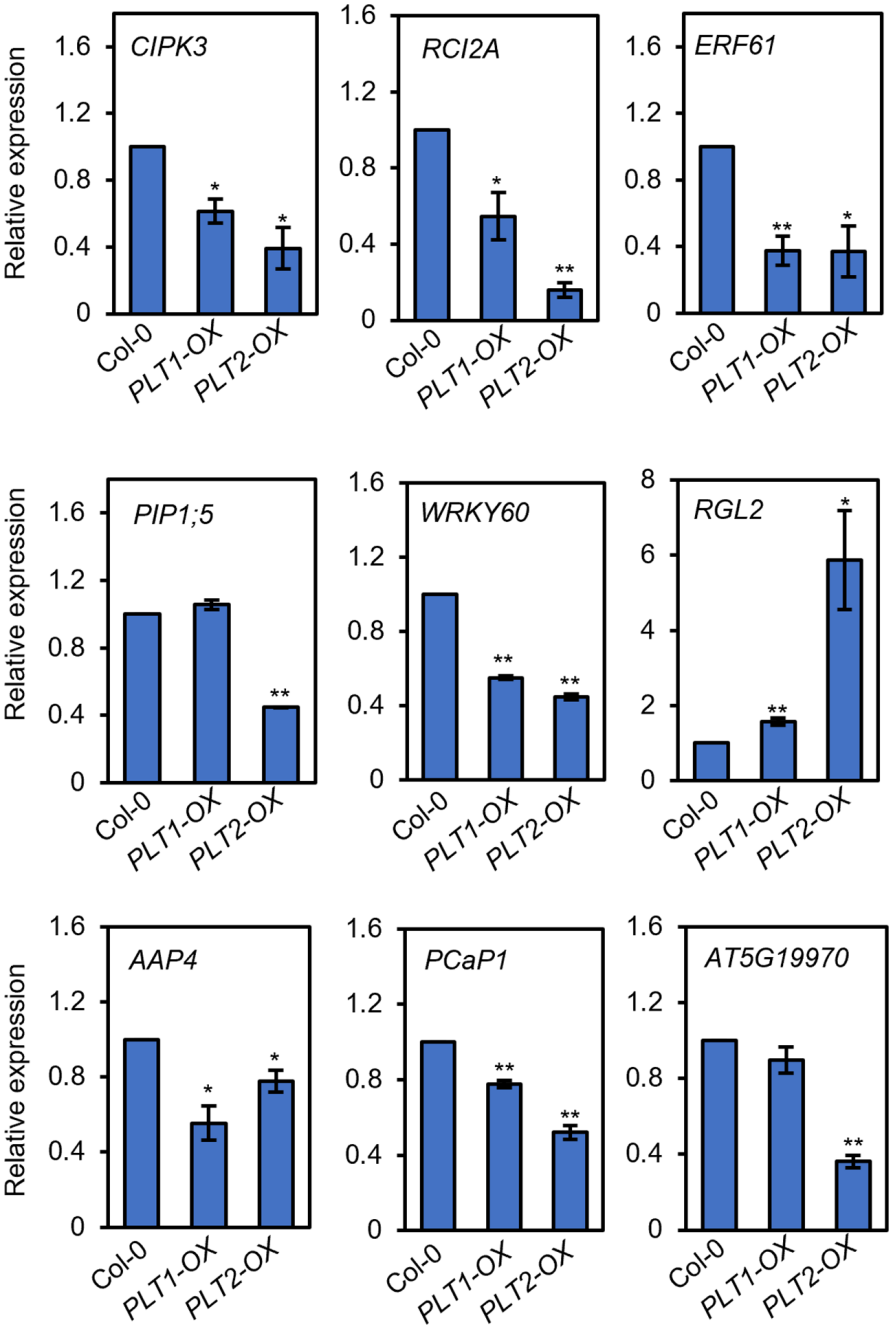
PLTs regulate the expression of drought-responsive genes. The relative expression levels of drought-responsive genes in roots of Col-0, *PLT1-OX*, and *PLT2-OX* plants. Error bars indicate the mean ± SD. Statistically significant differences are indicated by asterisks (Student’s *t*-test, *p < 0.05, **p < 0.01).

**Figure 8:**
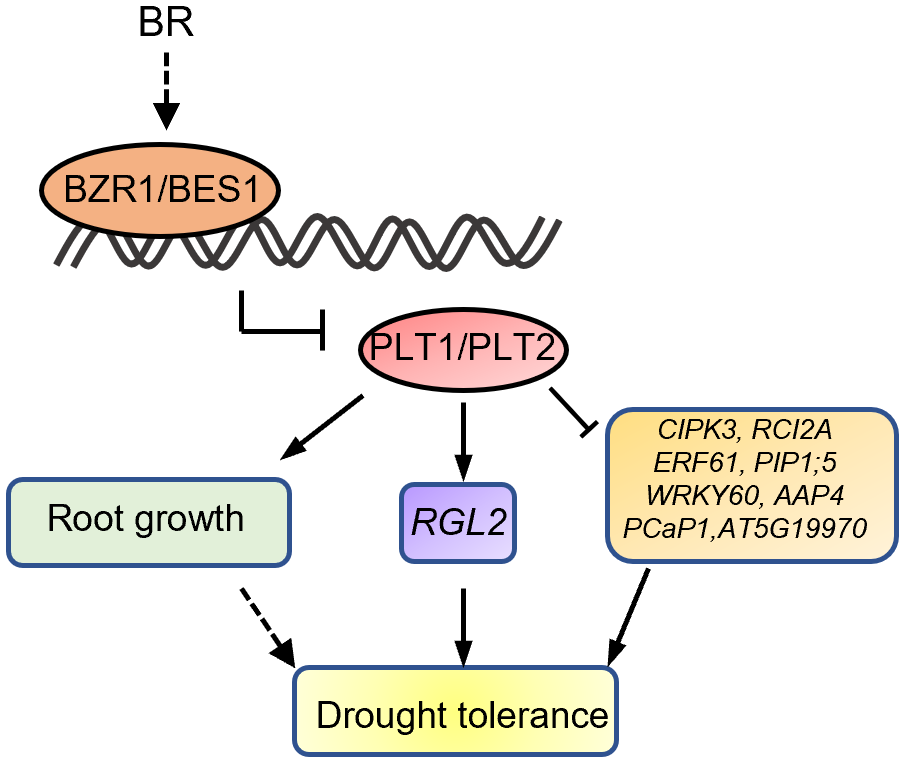
Proposed working model for the role of PLTs in BR signaling and drought stress.

To investigate the mechanism by which PLT1 and PLT2 regulate drought resistance in plants, we analyzed the downstream-regulated target genes of PLT1 and PLT2 by comparing the 3645 PLT1/2-targeted genes (Santuari et al., 2016) and 5310 BR-regulated Arabidopsis root genes (Chaiwanon and Wang, 2015) with 2900 drought-regulated genes (Wang et al., 2021) (**Supplementary Table S1**). We found 335 genes that were coregulated by BR, PLT1/2, and drought (**Figure 6A** and **Supplementary Table S2**). Further comparison of these 335 genes showed that 87 genes were downregulated by BR but were activated by PLT1/2; in contrast, 143 genes were upregulated by BR but were repressed by PLT1/2 (**Figure 6B**). GO analysis of these 230 genes showed enrichment in organic acid transport, chemical stimulation, abiotic stimulation, oxidative response, nitrogen transport, and hormone stimulation responses (including those for auxin, gibberellin, and abscisic acid [ABA]) (**Figure 6C** and **Supplementary Table S3**). We next searched for BR-regulated drought stress-related downstream genes *via* PLT1 and PLT2. We found by comparison that 19 of 87 BR-downregulated but PLT-activated genes were upregulated by drought, while 68 were downregulated by drought; in addition, 87 of 143 BR-upregulated but PLT-suppressed genes were upregulated by drought, while 56 were downregulated by drought (**Figure 6D**; **Supplementary Figure S3**, and **Supplementary Table S4**). Among these were several genes that are known to regulate the plant drought response, such as *CIPK3* (Sanyal et al., 2017), *RCI2A* (Medina et al., 2005), *PIP1;5* (Alexandersson et al., 2010), *ERF61* (Bano et al., 2022), and *WRKY60* (Chen et al., 2010; Arjmand et al., 2023). Through qRT−PCR, these genes were indeed found to be repressed in *PLT1-OX* and *PLT2-OX* (**Figure 7**). *PCaP1* is specifically expressed in endodermal cells of the root elongation zone and plays an important role in the hydrotropic response (Tanaka-Takada et al., 2019). *PCaP1* was downregulated by drought, repressed by PLT and upregulated by BR treatment and verified to be downregulated in *PLT1-OX* and *PLT2-OX* (**Figure 7**). *AAP4*, which is downregulated by dehydration, and *AT5G19970*, which is involved in hormone metabolic processes and root development, were repressed by PLT1/2 but upregulated by drought and BR treatment, and it was verified that these genes were indeed downregulated in *PLT1-OX* and *PLT2-OX* (**Figure 7**). *RGL2*, which encodes a DELLA protein that restrains the cell proliferation and expansion that drives plant growth, was upregulated by drought and PLT1/2 but downregulated by BR treatment, and it was verified that these genes were indeed upregulated in *PLT1-OX* and *PLT2-OX* (**Figure 7**). These findings suggest that in response to BR-mediated drought stress, the transcription factor PLT1/2 not only regulates the expression of genes corresponding to important drought or dehydration responses but also may regulate the expression of root growth and development genes and cell proliferation and expansion genes (**Figure 8**).

## 4 Discussion

Roots are the main organs that plants use to absorb water and nutrients. When drought occurs, the morphological structure of roots changes to enhance the water uptake capacity of plants. Longer roots with fewer branches are conducive to the survival of plants in a soil environment with dry surfaces but sufficient deep water. In contrast, shorter roots with more branches are more conducive to the survival of plants in areas with less rainfall (Dinneny, 2019; Gupta et al., 2020; Karlova et al., 2021). However, at present, we know very little about the molecular mechanisms by which drought regulates plant root morphogenesis.

Root length is an important factor in plant adaptation to drought and is significantly influenced by the activity of the root meristem (Beemster and Baskin, 1998). Several transcription factors and hormonal regulatory networks have been reported to play important roles in the development of the meristematic zone. Among them, WUSCHEL-RELATED HOMEOBOX-5 (WOX5), SHORT ROOT (SHR), SCARECROW (SCR), and PLTs are four important transcription factors/families that reportedly participate in regulating the development of the root meristem (Xu et al., 2020), but their relationship with drought stress has rarely been reported thus far. In this study, we screened different plants for drought sensitivity and found that *PLT1-OX* and *PLT2-OX* overexpression plants had elevated resistance to drought stress (**Figure 1**). Further comparison of PLT1 and PLT2 target genes and drought-regulated genes revealed 748 coregulated genes (**Figure 6A**). The drought-regulated genes *CIPK3*, *RCI2A*, *PIP1;5*, *ERF61*, and *WRKY60* were found to be repressed in *PLT1-OX* and *PLT2-OX* (**Figure 7**). These results suggest that PLT1 and PLT2, important transcription factors regulating root growth and development, are also involved in the drought stress response. This evidence expands the current understanding of the functions of PLT1/PLT2 and lays a foundation for further study of the relationship between root length and drought responses.

BR plays an important roles in Arabidopsis root growth and development; for example, the short-root phenotype is observed in the BR synthesis-deficient mutant *dwf4*, the signaling pathway-deficient mutant *bri1-116*, and the gain-of-function mutant *bes1-D* (Müssig et al., 2003; Kim et al., 2007; González-García et al., 2011; Chaiwanon and Wang, 2015), while *bzr1-1D-CFP* and *bzr1-1D* exhibit a partially alleviated short-root phenotype of *dwf4* and *bri1-116* (Chaiwanon and Wang, 2015). The transcription factors BZR1 and BES1 function mainly through transcriptional regulation. It has been reported that BZR1/BES1 can regulate meristem size by regulating the expression of *ERF115* and *BRAVO*, but it is unclear whether BZR1 and BES1 can also regulate the development of the root meristem by regulating other genes and how they affect the overall development of Arabidopsis roots.

In this study, we found that PLT1 and PLT2 are involved in the BR signaling pathway and that BZR1/BES1 represses the expression of *PLT1* and *PLT2* to regulate the development of the Arabidopsis root meristem. First, we found that BZR1 can bind to the promoter of *PLT1* and repress *PLT1* expression *in vivo*, which confirms that *PLT1* is a target gene of BZR1 (**Figure 2**). Second, we found that the expression of *PLT1* and *PLT2* in roots is repressed in response to BR treatment as well as in the BR gain-of-function mutants *bzr1-1D* and *bes1-D*, which indicates that the BR signaling pathway can indeed regulate the gene expression of *PLT1*/*PLT2* through the transcription factor BZR1/BES1 (**Figure 3**). Our results are consistent with previous reports that *PLT1* and *PLT2* expression is repressed after BR treatment (Chaiwanon and Wang, 2015; Vragović et al., 2015). Third, we found that compared with that of Col-0, the sensitivity of *PLT2-OX* to eBL was significantly decreased, while the sensitivity of *plt1 plt2* and *bzr1-1D* to eBL was enhanced, and the root meristem was disordered (**Figure 4**). This suggests that the role of PLT1/PLT2 in root growth and development is regulated by the BR signal transduction pathway and is genetically redundant. We have also previously found that SiBZR1 inhibits *SiPLT-L1* expression and affects root development in foxtail millet (Zhao et al., 2021), suggesting that the mechanism by which BRs regulate root elongation through PLTs is relatively conserved in both monocotyledons and dicotyledons. The receptor BRI1 recognizes BRs and conveys signals to the transcription factor BZR1/BES1. BZR1/BES1 binds directly to the promoter of *PLT1* and represses its expression, thereby regulating the development of the root apical meristem.

BR also plays a role in regulating plant responses to drought stress (Anwar et al., 2018; Nolan et al., 2019). Recent studies in Arabidopsis have shown that BRs inhibit the expression of RD26 and its homolog but promote the expression of *WRKY54* through BZR1/BES1, thereby negatively regulating the plant drought response (Chen et al., 2017; Ye et al., 2017). However, it has also been found that exogenous application of 1 μM eBL enhances drought tolerance in Arabidopsis (Kagale et al., 2007) and that the BR receptor BRL3 enhances drought tolerance in plants upon excessive accumulation in root vascular tissues (Fàbregas et al., 2018; Nolan et al., 2019). These conflicting results suggest that BR may affect plant drought tolerance in a tissue-specific manner through multiple regulatory mechanisms, but the molecular mechanisms involved need to be further explored. In this study, we mainly investigated whether PLTs are involved in BR-mediated drought stress response, but since BZRs family transcription factors, which had six paralog genes in the Arabidopsis genome, and they function redundantly in regulating Arabidopsis development and BR signaling. No obvious phenotype can be observed for BZRs single mutants, double mutants and quadruple mutant (Chen et al., 2019). Unfortunately, it is hard for us to construct the *bzr1 bes1 beh1 beh2 beh3 beh4 plt1 plt2.* Therefore, we used the gain-of-function mutant *bes1-D* and *PLTs* overexpression plants for the study. We found that *PLT1-OX* and *PLT2-OX* plants had enhanced tolerance to drought stress (**Figure 1**) and that *PLT2* overexpression alleviated the *bes1-D* drought-sensitive phenotype (**Figure 5**). These results suggest that BR can negatively regulate plant drought tolerance by suppressing the expression of *PLTs* through BZR1/BES1. Our results identify new target genes of BR, PLT1/PLT2, in the drought stress response. However, *PLT2-OX* only partially recovered the drought sensitive phenotype of *bes1-D* might be that *bes1-D* regulates a number of downstream target genes, including PLT1/2, and then regulates plant drought responses. Whether PLTs can interact with RD26 or WRKY54 to regulate drought tolerance in plants and the regulatory relationship between PLTs and the receptors BRI1 and BRL3 need to be further investigated.

Taken together, our findings thus far revealed that BR can regulate root growth and plant responses to drought through PLTs. However, how does BZR1/BES1-PLT affect plant drought tolerance? Does BZR1/BES1-PLT respond to drought stress by regulating root morphogenesis? To answer these questions, we performed a comprehensive analysis by comparing the downstream-regulated target genes of PLT1 and PLT2 (Santuari et al., 2016), BR-regulated Arabidopsis root genes (Chaiwanon and Wang, 2015), and drought-regulated genes (Wang et al., 2021). First, the analysis revealed that BR and PLTs coregulated the expression of some drought-related genes in opposite ways (**Figure 6** and **Supplementary Figure S3**), and we selected some of them for validation. We found that the expression levels of *CIPK3*, *RCI2A*, *PIP1;5*, *ERF61*, *AAP4*, and *WRKY60* were suppressed by PLT1/2, while that of *RGL2* was enhanced by PLT1/2 (**Figure 7**). The results suggest that BZR1/BES1-PLTs may participate in drought stress control by regulating the expression of downstream drought-responsive genes. Moreover, we found that *PCaP1* and *AT5G19970*, which are involved in root development, were downregulated by PLT1/2 (**Figure 7**). We also focused on 75 genes that were coregulated by drought, PLTs, and BR treatment. We found that they were significantly enriched in root morphogenesis (**Supplementary Figure S3**), and many of them were genes that are known to regulate root development and are related to the auxin signaling pathway. This indicates that BR and auxin may engage in crosstalk in root morphogenesis under drought conditions. In addition, *PIP1;5* and *AT5G19970* were downregulated in PLT2-OX, while there were no significant changes in PLT1-OX (**Figure 7**). This may have been due to the specificity of PLT1 and PLT2 in the regulation of homologous gene families of downstream target genes, such as the downregulation of *SiPIP2;1* and *SiPIP2;2* mediated by the PLT1-homologous gene SiPLT-L1 in foxtail millet (Zhao et al., 2021).

## 5 Conclusions

In conclusion, our results suggest that after BR treatment, the expression levels of *PLTs* in roots are reduced, the root system is weak, and water uptake ability is reduced, resulting in drought sensitivity. However, whether BZR1/BES1-PLTs can regulate plant drought tolerance by increasing plant root length and the root phenotypes of related plants under drought conditions, especially their phenotypes in soil, still need to be further studied. Such research will enhance understanding of how BRs mediate root elongation under drought and will be necessary to improve the drought resistance of crops in the future.

## Data availability statement

The original contributions presented in the study are included in the article/**Supplementary Material**. Further inquiries can be directed to the corresponding authors.

## Author contributions

WT and BZ designed research; ZZ performed most of the research; W and HG performed the qRT-PCR and genetic experiment; ZZ, XW and BZ wrote the manuscript. All authors have read and approved the final manuscript.
