# Supplementary material for: The BR signaling pathway regulates primary root development and drought stress response by suppressing the expression of PLT1 and PLT2 in Arabidopsis thaliana
Source: Front Plant Sci. 2023 Jun 27;14:1187605. doi: 10.3389/fpls.2023.1187605 (PMC10333506; doi:10.3389/fpls.2023.1187605)
Supplement: Supplementary file 1 [file Presentation_1.pptx]

## Slide 1
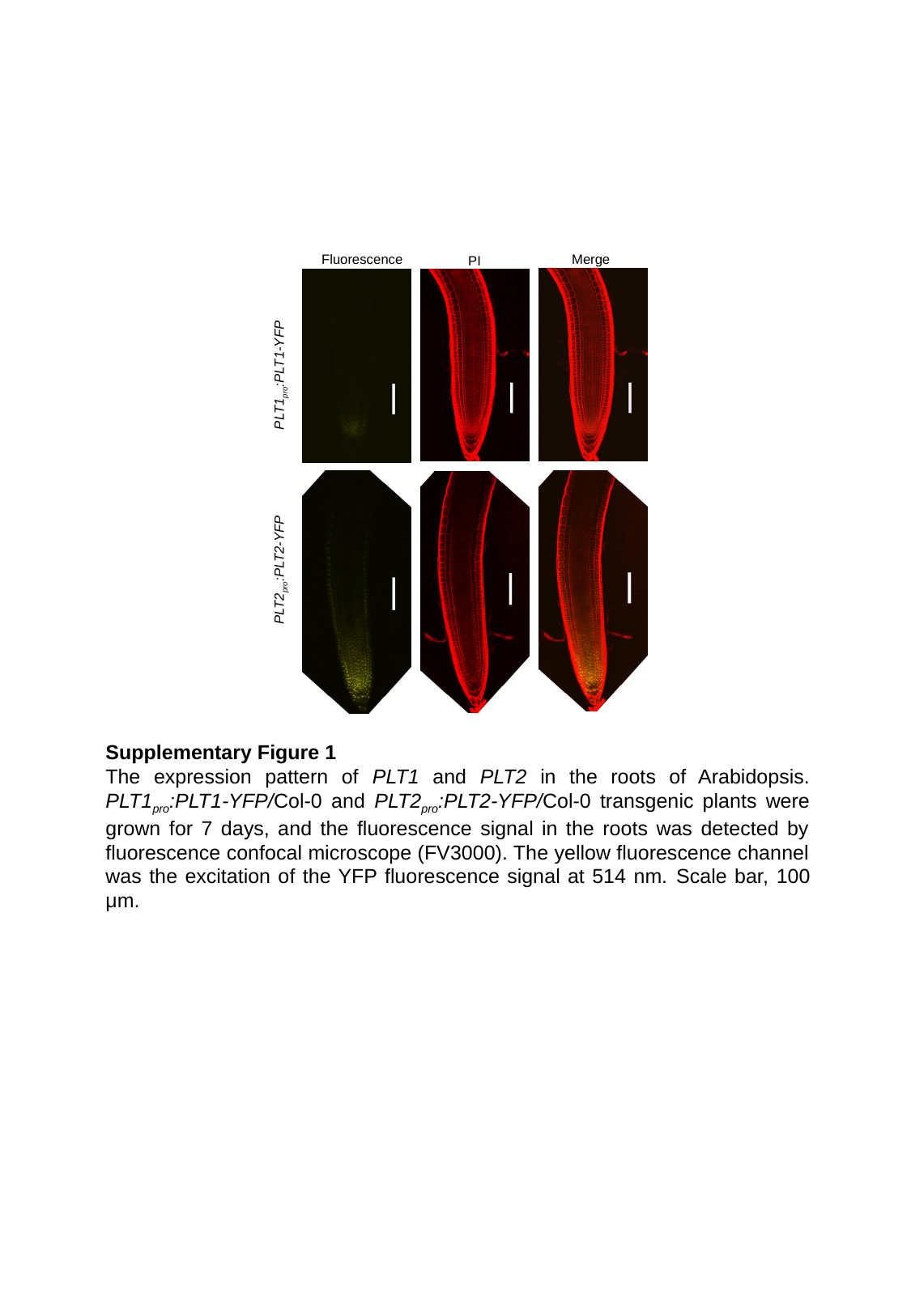

Fluorescence
Merge
PI
PLT2pro:PLT2-YFP
PLT1pro:PLT1-YFP
Supplementary Figure 1
The expression pattern of PLT1 and PLT2 in the roots of Arabidopsis. PLT1pro:PLT1-YFP/Col-0 and PLT2pro:PLT2-YFP/Col-0 transgenic plants were grown for 7 days, and the fluorescence signal in the roots was detected by fluorescence confocal microscope (FV3000). The yellow fluorescence channel was the excitation of the YFP fluorescence signal at 514 nm. Scale bar, 100 μm.

## Slide 2
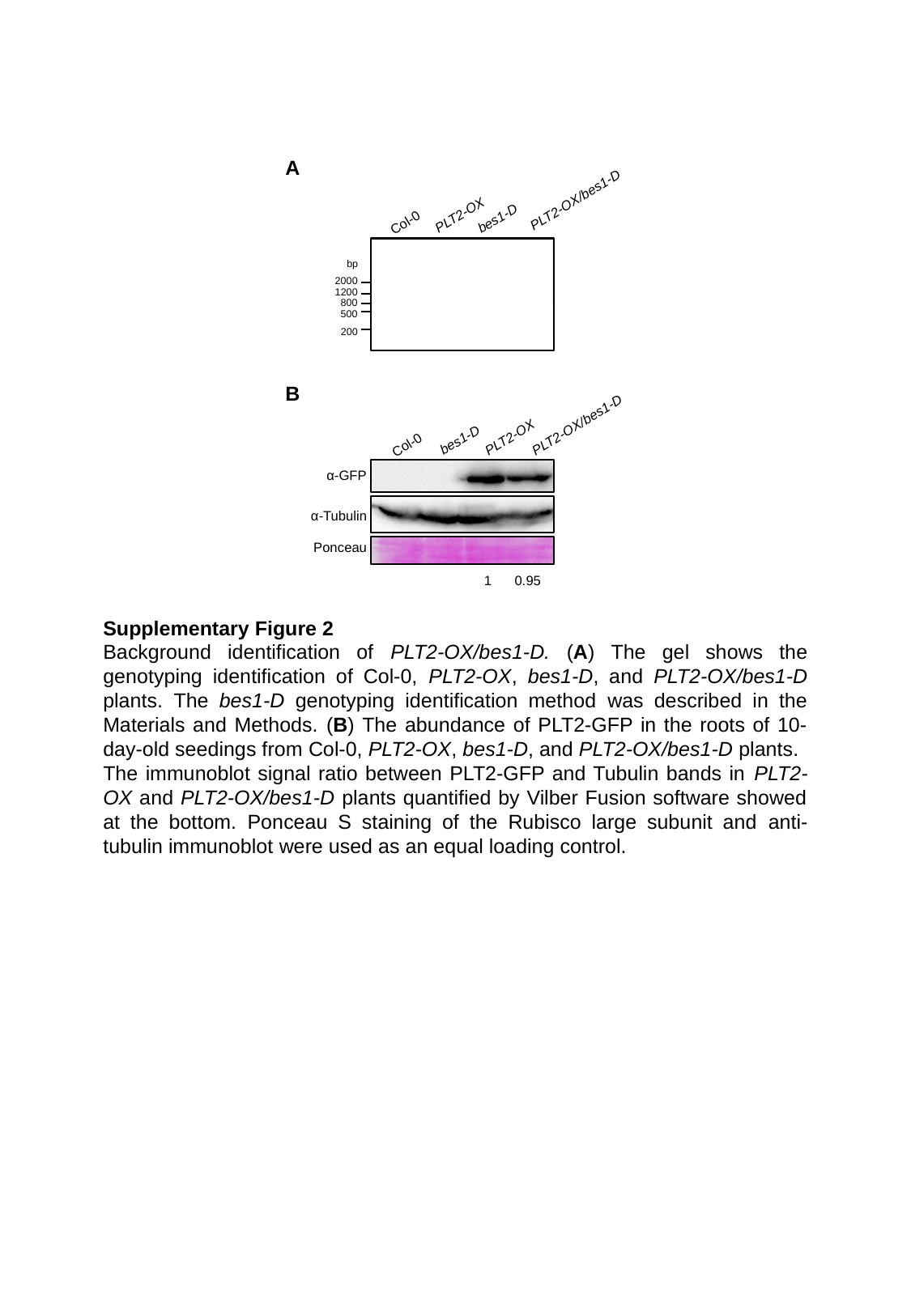

A
PLT2-OX/bes1-D
PLT2-OX
bes1-D
Col-0
bp
2000
1200
800
500
200
B
PLT2-OX/bes1-D
bes1-D
PLT2-OX
Col-0
α-GFP
α-Tubulin
Ponceau
1 0.95
Supplementary Figure 2
Background identification of PLT2-OX/bes1-D. (A) The gel shows the genotyping identification of Col-0, PLT2-OX, bes1-D, and PLT2-OX/bes1-D plants. The bes1-D genotyping identification method was described in the Materials and Methods. (B) The abundance of PLT2-GFP in the roots of 10-day-old seedings from Col-0, PLT2-OX, bes1-D, and PLT2-OX/bes1-D plants.
The immunoblot signal ratio between PLT2-GFP and Tubulin bands in PLT2-OX and PLT2-OX/bes1-D plants quantified by Vilber Fusion software showed at the bottom. Ponceau S staining of the Rubisco large subunit and anti-tubulin immunoblot were used as an equal loading control.

## Slide 3
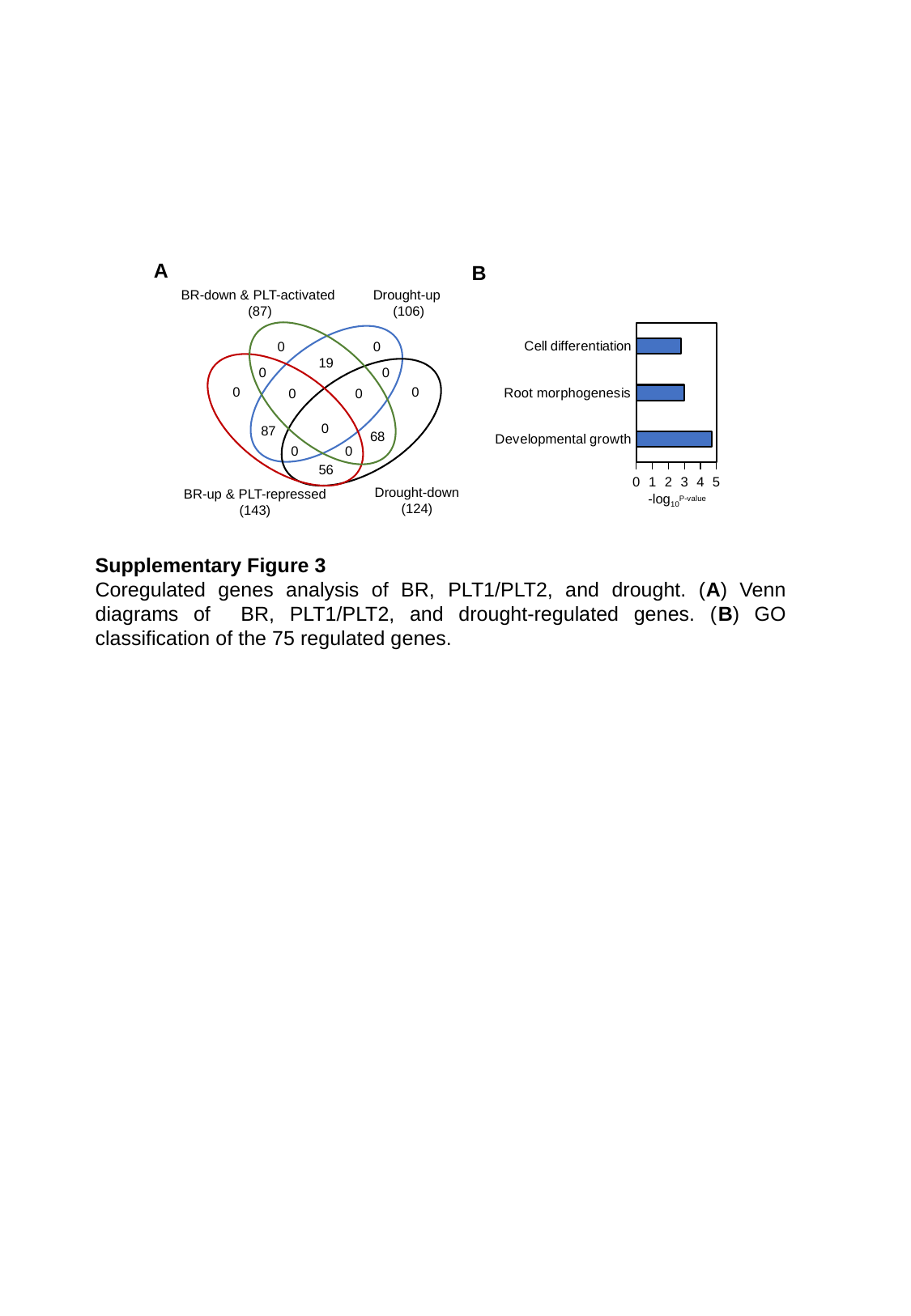

A
B
BR-down & PLT-activated
(87)
Drought-up
(106)
### Chart
| Category | |
|---|---|
| Developmental growth | 4.711944359980624 |
| Root morphogenesis | 3.005557027432874 |
| Cell differentiation | 2.7859482250823278 |-log10P-value
0
0
19
0
0
0
0
0
0
0
87
68
0
0
56
Drought-down
(124)
BR-up & PLT-repressed (143)
Supplementary Figure 3
Coregulated genes analysis of BR, PLT1/PLT2, and drought. (A) Venn diagrams of BR, PLT1/PLT2, and drought-regulated genes. (B) GO classification of the 75 regulated genes.
